# Clinical and Genetic Characterization of Patients with Primary Ciliary Dyskinesia in Southwest Saudi Arabia: A Cross Sectional Study

**DOI:** 10.3390/children10101684

**Published:** 2023-10-13

**Authors:** Ali Alsuheel Asseri, Ayed A. Shati, Ibrahim A. Asiri, Reem H. Aldosari, Hassan A. Al-Amri, Mohammed Alshahrani, Badriah G. Al-Asmari, Haleimah Alalkami

**Affiliations:** 1 Department of Child Health, College of Medicine, King Khalid University, Abha 62529, Saudi Arabia; ashati@kku.edu.sa; 2 Departments of Pediatrics, King Khalid University Medical City, Abha 62223, Saudi Arabia; ibasiri@kku.edu.sa; 3 College of Medicine, King Khalid University, Abha 62529, Saudi Arabia; 438800377@kku.edu.sa; 4 Department of Pediatrics, Khamis Mushayt Children Hospital, Khamis Mushayt 62454, Saudi Arabia; halamri6@moh.gov.sa; 5 Department of Pulmonology, Aseer Central Hospital, Abha 62523, Saudi Arabia; malshahrani24@moh.org.sa; 6 Department of Pediatrics, King Fahad Military Hospital, Khamis Mushayt 31932, Saudi Arabia; b-a-1985@hotmail.com; 7 Department of Pediatrics, Abha Maternity & Children Hospital, Abha 3613, Saudi Arabia; haalalkami@moh.gov.sa

**Keywords:** children, primary ciliary dyskinesia, bronchiectasis, DNAH5, RSPH9, Saudi Arabia

## Abstract

Background: Primary ciliary dyskinesia (PCD, MIM 244400) is an inherited ciliopathy disorder characterized by recurrent sinopulmonary infections, subfertility, and laterality defects. The true incidence of PCD in Saudi Arabia is not known, but it is likely underdiagnosed due to the high prevalence of consanguineous marriages. In this study, we aim to study the clinical and genetic characteristics of PCD patients in the southwestern region of Saudi Arabia to provide guidance to clinicians and researchers studying PCD. Methods: This was a cross-sectional study conducted between 2019 and 2023 in Abha Maternity and Children’s Hospital. Twenty-eight patients with clinically diagnosed PCD were recruited. The diagnosis of PCD was confirmed via whole-exome sequencing. Results: A total of 28 patients from 20 families were identified and recruited for this study. The median age of patients was 7.5 years (IQR = 3, 13 years). The people of different sexes were evenly distributed, and 18 patients (64%) had neonatal respiratory distress (NRD). The median age of diagnosis was 5.5 years (IQR = 2, 11 years), while the age when the first symptoms appeared was 3 months old (IQR = 1, 6 months). The prevalence of a chronic wet cough, chronic rhinosinusitis, ear infections were 100% (n = 28), 78.6% (n = 22), and 67.9% (19), respectively. The most common gene in our study was DNAH5, which represented 17.9% (five out of twenty-eight) of the cases. Furthermore, the remaining pathogenic variants included: 14.3% with RSPH9 in four individuals (three families), 14.3% with DNAI2 in four individuals (two families), and 10.7% with LRRC56 in three individuals (one family). The most common findings on the chest CT scans were consolidation (seen in all patients), mucus plugging (seen in 95%), and bronchiectasis (seen in 77%). In the patients with bronchiectasis, the most commonly affected lobes were the right lower lobe (88%) and left lower lobe (76%). The patients with PCD and situs inversus were more likely to experience NRD than the patients with PCD and situs solitus. The median PICADAR score in the patients with PCD and situs inversus (median: 11.5; Q1: 10–Q3: 12.5) was significantly higher compared to those with PCD and situs solitus (median: 7.5; Q1: 5.8–Q3: 8) (U = 10.5; *p* < 0.001). Conclusion: This study provides preliminary data on the clinical and genetic characteristics of PCD patients in the southwestern region of Saudi Arabia. We found that DNAH5 and RSPH9 genes were the most common genes among the studied population. Furthermore, PCD should be considered for each child with early NRD and laterality defects, and further confirmatory tests are recommended. These findings also highlight the need for greater awareness of the disease in daily clinical practice to facilitate early diagnosis and avoid irreversible lung damage.

## 1. Introduction

Primary ciliary dyskinesia (PCD, MIM 244400) is an inherited motile ciliopathy disorder characterized by recurrent sinopulmonary infections, subfertility, and laterality defects [1]. PCD is most commonly inherited in an autosomal recessive pattern, but autosomal dominant and X-linked recessive patterns have also been reported [2]. It is individually rare, but is collectively common, with huge impact on the patients’ quality of life as well as morbidity and mortality [3,4,5,6]. Although PCD has been reported in many ethnic groups, the true prevalence is still unknown [7,8,9]. Based on a large international survey of European pediatric PCD patients, the estimated prevalence ranged from 1:10,000 to 1:20,000 live-born children. The true incidence of PCD in Saudi Arabia (SA) is not known, but it is likely underdiagnosed due to the high prevalence of consanguineous marriages [10].

The diagnosis of PCD can be delayed or missed until adulthood due to a lack of awareness of the disease and/or the difficulty in confirming it. It has a spectrum of clinical manifestations that start in the first year of life and progress with time, leading to bronchiectasis and respiratory failure [3,4,11]. The official clinical guidelines of the American Thoracic Society (ATS) for PCD diagnosis state that a further PCD diagnostic work up for patients with two out of the following four criteria is recommended: unexplained neonatal respiratory distress (NRD) in term infants, year-round daily cough beginning before 6 months of age, year-round daily nasal congestion beginning before 6 months of age, and organ laterality defects [12]. Although PCD can manifest in early infancy, the confirmation of the diagnosis is challenging, even in highly specialized PCD centers, due to the lack of a gold-standard test. Based on the North American PCD Foundation, about 10% of people with PCD have been definitively diagnosed and followed up with at a PCD clinical center [13]. 

Several functional assay tools are used for PCD diagnosis, such as transmission electron microscopy (TEM), high-speed video microscopy, and nasal nitric oxide; however, these tools are complex, expensive, and require special expertise [12,14]. For several years, TEM has been used as the gold-standard diagnostic modality for PCD diagnosis; nevertheless, TEM data are normal in approximately 30% of PCD cases [12,13]. In recent years, there has been growing interest in using genetic testing as the first modality to confirm the diagnosis of PCD in people with a compatible PCD phenotype, especially when other functional tests are not available [13,15]. An ever-increasing body of literatures shows that over 50 genes have been linked to PCD, and the genetic understanding of PCD is evolving as new genes are discovered [13,16,17]. Although there is no cure for PCD, early diagnosis and management are essential for improving long-term lung function [5]. The standard therapy for PCD currently includes regular airway clearance and aggressive antibiotic therapy for pulmonary exacerbations, which is mostly extrapolated from diseases such as cystic fibrosis (CF). However, a proper definition of the PCD phenotype and genotype may introduce the possibility of discovering future mutation-specific therapies, as occurred in CF. Herein, we aim to study the clinical and genetic characteristics of PCD patients in the southwestern region of Saudi Arabia to provide guidance to clinicians and researchers studying PCD.

## 2. Materials and Methods

### 2.1. Patients and Study Setting

This was a cross-sectional study conducted between 2019 and 2023 in Abha Maternity and Children’s Hospital. Twenty-eight patients with clinically diagnosed PCD were recruited. Clinical PCD diagnoses were made based on the latest ATS diagnostic guidelines, which require the presence of two out of four of the following criteria: (a) a persistent, wet cough that starts in the first 6 months of life, (b) persistent, nasal congestion that starts in the first 6 months of life, (c) the presence of organ laterality abnormalities, and (d) unexplained NRD in infants born at term gestation [12]. Additionally, all patients completed the PICADAR questionnaire. PICADAR is a clinical score that evaluates the presence of certain symptoms and signs of PCD, such as a daily wet cough that started in infancy, unexplained chest symptoms in the neonatal period, neonatal intensive care admission, situs abnormality, congenital heart disease, persistent year-round rhinitis, and chronic ear or hearing symptoms. PICADAR is a simple clinical prediction score with sensitivity of 90% for a cut-off score of 5 points [18]. Clinical and radiological data were collected from the patients and their caregivers, as well as from their medical records. These data included medical history, physical examination, and imaging findings. A diagnosis of PCD was confirmed by finding two mutations in one or two genes that are known to cause PCD. The diagnosis of chronic rhinosinusitis was based on the presence of 2 or more symptoms of purulent rhinorrhea, nasal obstruction, facial pressure/pain, or a cough and either endoscopic signs of mucosal edema, purulent drainage, or nasal polyposis and/or paranasal sinuses computed tomography (CT) scan changes showing mucosal changes within the ostiomeatal complex and/or sinuses including sinus mucosal thickening, a sinus ostial obstruction, and sinus opacification [19].

### 2.2. Genetic Testing

The diagnosis of PCD was confirmed via whole-exome sequencing (WES) for all the patients. All molecular genetic studies were performed in accredited commercial laboratories, such as CENTOGENE (https://www.centogene.com/, accessed on 1 March 2022) and PerkinElmer Genomics (https://www.perkinelmergenomics.com/, accessed on 20 August 2022). Deoxyribonucleic acid (DNA) was extracted using a PerkinElmer Chemagic DNA CS200 DNA extraction kit (PerkinElmer, Waltham, MA, USA) with the Chemagic 360 instrument (PerkinElmer, Waltham, MA, USA). The DNA quality was checked and quantified with the PicoGreen reagent (ThermoFisher, Waltham, MA, USA) and an Enspire plate reader (PerkinElmer, Waltham, MA, USA). Whole-exome sequencing was performed on the genomic DNA using the Agilent SureSelect Clinical Research Exome v3 targeted sequence capture method to enrich for the exome following standard protocols [20]. The direct sequencing of the amplified captured regions was performed using 2 × 150 bp paired end reads on NovaSeq 6000 Illumina next-generation sequencing (NGS) systems. The detailed method for WES has been published previously [21]. 

### 2.3. Radiological Image Acquisition

All the patients underwent chest radiography (CXR) using a Carestream (Carestream Health, Rochester, NY, USA) machine at their first visits. In addition, 17 patients underwent high-resolution chest CT and CT scans of the paranasal sinuses using a 16-slice CT scanner (Siemens Healthcare, Erlangen, Germany). The indications for a chest CT scan were recurrent pulmonary exacerbations and the presence of signs of chronic suppurative lung diseases, such as finger clubbing and chest crackles. CXR and CT films were interpreted by a pediatric radiologist with ten years of experience following passing the medical board exam. CXR and CT films were evaluated for the presence, distribution, and characteristics of consolidation, mucus plug, and bronchiectasis. Changes in the paranasal sinuses on CT scan were assessed for mucosal thickening, the obstruction of the sinus ostia, and the opacification of the sinuses.

### 2.4. Statistical Analysis

Statistical analysis was performed using Stata version 14 (StataCorp, College Station, TX, USA). Non-normally distributed variables were summarized as median (interquartile range, IQR). Categorical variables were summarized as counts and percentages. The non-parametric Mann–Whitney U test was used to compare non-normally distributed continuous variables. The chi-square test was used to compare categorical variables. A *p*-value of less than 0.05 was considered statistically significant.

## 3. Results

### 3.1. Clinical Phenotyping of the Study Population

**Figure 1:**
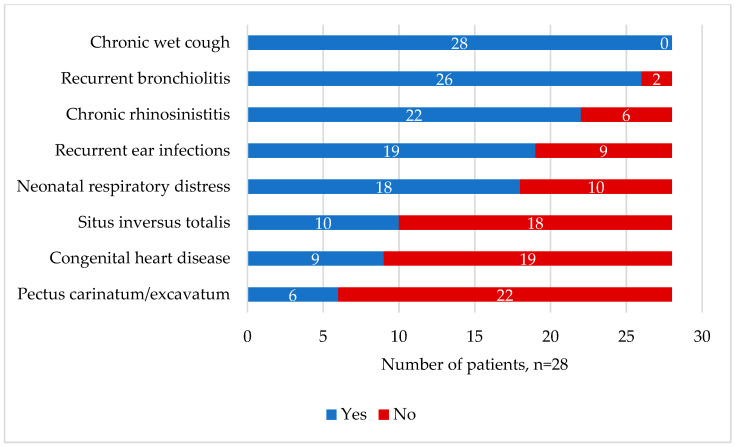
Clinical information on signs, symptoms, and comorbidities of the enrolled patients (n = 28). Congenital heart disease was diagnosed based on the echocardiogram results.

A total of 28 patients from 20 families were identified and recruited for this study. The baseline clinical characteristics, including neonatal history, presenting symptoms, and growth parameters, are summarized in Table 1. The median age of patients was 7.5 years (IQR = 3, 13 years). The patients of different sexes were evenly distributed, and 18 patients (64%) had NRD and required NICU admission, with a median hospital stay duration of 18 days (IQR = 11, 42 days). The median age of diagnosis was 5.5 years (IQR = 2, 11 years), while the age when the first symptoms appeared was 3 months old (IQR = 1, 6 months). Seven patients (25%) had a sibling with a confirmed diagnosis of PCD, and sixteen patients (57.1%) had a family member with suspected PCD. The consanguinity rate among the enrolled study participants was 93%. The median BMI percentile (%) was 12% (IQR = 3.5, 46th %), and eight patients (28.6%) had a BMI % below the 5th percentile. The median PICADAR score was eight (IQR = 6,12). The prevalence of chronic wet coughs, chronic rhinosinusitis, ear infections were 100% (n = 28), 78.6% (n = 22), and 67.9% (19), respectively. Of the 28 patients with PCD, 64% had situs solitus, and 36% had situs inversus. Overall, 32% of the patients had congenital heart disease, and 21% had pectus excavatum/carinatum (Figure 1).

### 3.2. Genetics of the Study Population

**Figure 2:**
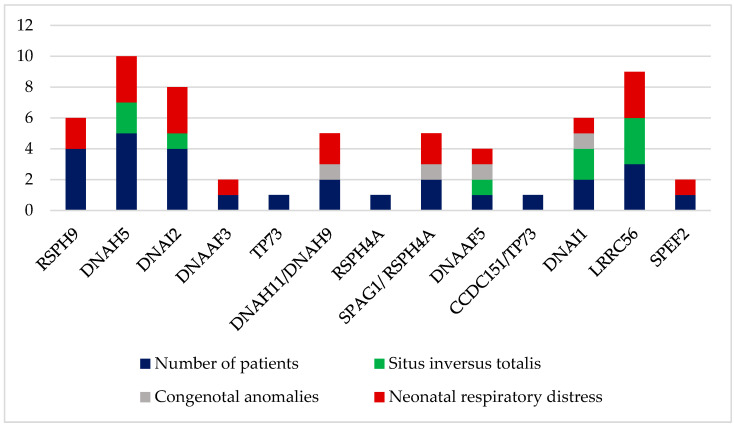
Characteristics of primary ciliary dyskinesia genes in the enrolled patients (n = 28). DNAH5 was the most prevalent. Blue color indicates the number of patients in particular gene. Green color indicates the number of patients with situs inversus totalis. Grey color indicates the number of patients with congenital anomalies. Red color indicates the number of patients with neonatal respiratory distress.

Table 2 and Figure 2 show the genetic results and the associated phenotypes of the enrolled patients. The most common gene in our study was DNAH5, which represented 17.9% (five out of twenty-eight) of the cases. Furthermore, the remaining pathogenic variants included: 14.3% with RSPH9 in four individuals (three families), 14.3% with DNAI2 in four individuals (two families), 10.7% with LRRC56 in three individuals (one family), 7.1% with DNAI1 in two individuals (one family), 7.1% with DNAH11/DNAH9 in two individuals (two families), 7.1% with SPAG1/RSPH4A in two individuals (one family), 3.6% with SPEF2 in one individual (one family), 3.6% with CCDC151/TP73 in one individual (one family), 3.6% with DNAAF5 in one individual (one family), 3.6% with RSPH4A in one individual (one family), 3.6% with TP73 in one individual (one family), and 3.6% with DNAAF3 in one individual (one family). The genes that were linked to situs inversus totalis include DNAH5, DNAI2, DNAAF5, DNAI1, and LRRC56. Four patients of our study subjects had additional associated anomalies that included hepatic hemangioendothelioma, anorectal malformation, congenital aplastic anemia, and congenital asplenia: the patients’ numbers were 21, 9, 19, and 23, respectively.

### 3.3. Radiological Findings of the Study Population

**Figure 3:**
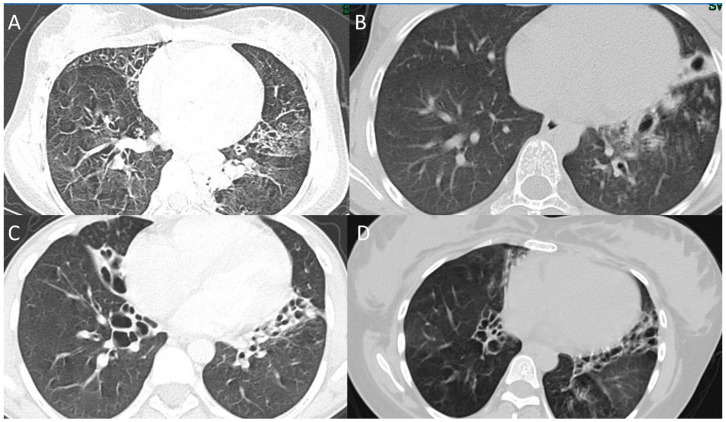
CT scans chest findings (patients 17, 12, 16, and 7). (**A**) CT scan of patient 17 (14-year-old female with RSPH9 gene mutation) shows mosaics pattern with bilateral bronchiectasis. (**B**) CT scan of the patient 13 (13-year-old female with TP73 gene mutation) shows left lower lobe cystic bronchiectasis. (**C**) CT scan of the patient 16 (15-year-old male with DNAH5 gene mutation) shows bilateral cystic bronchiectasis. (**D**) CT scan of the patient 8 (25-year-old female with DNAH5 gene mutation) shows mosaic attenuation with bilateral bronchiectasis.

The CXR and CT chest findings of the enrolled patients are summarized in Table 3. CXRs were conducted on all 28 patients, and chest CTs were performed on 17 of them. The CXRs showed lobar collapse/consolidation in 19 (67.9%) and peribronchial wall thickening in 24 (85.7%) of the patients. The most common findings on chest CT scans were consolidation (seen in all patients), mucus plugging (seen in 95%), bronchiectasis (seen in 77%), and ground-glass density (seen in 36%). In patients with bronchiectasis, the most commonly affected lobes were the right lower lobe (88%), left lower lobe (76%), right middle lobe (65%), left upper lobe (47%), and right upper lobe (29%). Selected CT chest findings for some of the enrolled patients are shown in Figure 3.

### 3.4. Characteristics of PCD in Patients with and without Situs Inversus

Table 4 reveals the characteristics of PCD in the patients with situs solitus and situs inversus. The PCD patients with situs inversus compared to those with PCD with situs solitus did not differ in terms of age at the time of the study, age at the time of the diagnosis, duration of NICU admission, BMI percentile, and the prevalence of bronchiectasis. However, the median age of symptom onset was slightly higher in the patients with PCD and situs solitus (median: 4 months; Q1: 2–Q3: 7 months) than it was in the patients with PCD and situs inversus (median: 1 month; Q1: 0–Q3: 5 months). This difference was marginally significant (Mann–Whitney U test, U = 55, *p* = 0.090). A chi-square test was performed to examine the association between NRD and laterality defects (situs inversus and situs solitus). The association between these variables was significant; X^2^ (1, n = 28) = 4.5, *p* = 0.040. The patients with PCD and situs inversus were more likely to experience NRD than the patients with PCD and situs solitus were. The median PICADAR score in the patients with PCD and situs inversus (median: 11.5; Q1: 10–Q3: 12.5) was significantly higher compared to those with PCD and situs solitus (median: 7.5; Q1: 5.8–Q3: 8) (U = 10.5; *p* < 0.001).

## 4. Discussion

Since the discovery of the first PCD-associated gene in 2000 [22], PCD has remained a heterogeneous disease with poor phenotype–genotype correlation [23]. Despite the symptoms of PCD appearing in early infancy [14,24], diagnosis is often delayed [1,25,26,27]. Until recently, TEM was the gold-standard diagnostic test for PCD, but it was only available in a few specialized centers. Herein, we aimed to study the clinical and genetic features of patients with PCD in the southwestern region of Saudi Arabia. To our knowledge, this is the first study to describe the clinical and genetic features of PCD patients in this region. Twenty-eight patients with classic symptoms of PCD and evidence of two mutations in genes associated with PCD were reported. The genes involved were CCDC151, DNAAF3, DNAAF5, DNAH11, DNAH5, DNAH9, DNAI1, DNAI2, LRRC56, RSPH4A, RSPH9, SPAG1, SPEF2, and TP73. These genes are essential to produce proteins that are important for the structural and functional integrity of cilia. The proteins they produce include components of the outer dynein arms ((ODA) and (DNAI1, DNAI2, DNAH5, DNAH9, and DNAH11)), a component of the ODA docking complex (CCDC151), an assembly of ODA and IDA complexes (DNAAF3, SPAG1, and DNAAF5), a component of the central sheath (SPEF2), components of the radial spoke (RSPH4A and RSPH9), and the absence of ODA in a distal portion of the axoneme (LRRC56) [1]. The phenotype of these gene defects is largely determined by the respiratory manifestations, which are present at all ages.

Although our findings revealed that the symptoms can appeared as early as 3 months of age, the median age of diagnosis was 5.5 years. In accordance with the present results, previous studies have demonstrated that mean age of PCD diagnosis was 4.4–6 years [24,28]. A delay in diagnosis can have a significant impact on the patients’ long-term pulmonary manifestation, as it can lead to decline in lung function and bronchiectasis [3]. The explanation for delayed diagnosis is likely due to the overlapping between PCD symptoms and other diseases such as asthma, protracted bacterial bronchitis, CF, and aerodigestive disorders. Additionally, the lack of awareness of this disease could contribute to this diagnosis delay. In this study, we followed the latest ATS diagnostic guidelines, which require the presence of PCD features before ordering a confirmation test. We found that the prevalence of a chronic wet cough, chronic rhinosinusitis, ear infections, and situs inversus was 100%, 78.6%, 67.9%, and 36%, respectively. Furthermore, 18 patients (64%) had NRD. The findings of our study corroborate the existing literature that state that these manifestations are prevalent in PCD patients [1,8,29,30]. The findings of our study are in line with those of Alzaid et al. (2021), who also found that all 18 enrolled patients with PCD reported a history of chronic coughs and chronic sinusitis [31]. Notably, the prevalence of consanguineous marriages in this population was high, at 93%. It is worth noting that consanguinity is common in the Saudi population [10], which could partly explain the high prevalence of autosomal recessive disorders [32]. The findings of this study suggest that the actual prevalence of PCD, especially in populations with high levels of consanguinity, is underestimated and could be higher than previously thought compared to those of other countries that have PCD registries and estimated PCD prevalence levels. Therefore, it is essential to increase the awareness among clinicians about the PCD symptoms and the importance of early diagnosis, with the highest level of awareness needed among healthcare workers at the primary healthcare level.

PCD is one of the airway clearance disorders that is caused by ciliopathy, which involve multiple organs or systems, especially in the respiratory tract [15,30]. So far, over 50 genes have been reported as disease-causing for PCD [15]. In our study, the DNAH5 gene, which encodes a dynein protein that is part of a microtubule-associated motor protein complex, was the most common gene. This finding is consistent with the findings of other studies in this area, which have also reported that DNAH5 is the most common gene in PCD patients. Zhao et al. (2021) found that DNAH5 was the most prevalent disease-causing gene in 23.1% of 26 patients with PCD of Chinese origin [8]. Moreover, DNAH5 was also the most prevalent gene among a PCD cohort from Turkey that represented 26.1% (12 out of 46 individuals) [25]. On the contrary, two recent studies reported from Saudi Arabia found that RSPH9 was the most common gene identified [31,33]. Furthermore, our finding is contrary to previous studies which have found that CCDC39 and CCDC40 are the most prevalent mutated genes in individuals with PCD of Egyptian and Tunisia origin [34,35]. The results of these studies, including our own, should be interpreted with caution due to the small sample sizes and the fact that they were all conducted in tertiary hospitals. These factors may have biased the results and preventing the accurate estimation of these genes prevalence among all PCD patients. Additionally, the occurrence of genetic variations among these studies may add to the complexity of PCD genetics in the Arab population. This necessitates further collaboration between healthcare centers and pediatric pulmonologists to promptly identify PCD patients and provide appropriate counseling.

In our study cohort, 17 out of 22 patients who had a chest CT scan had bronchiectasis, and all of them were over 8 years old. Furthermore, our results demonstrated that lower lobes were the most commonly involved lobes. In contrast to CF, data from several studies have revealed that the structural lung damage in PCD patients starts later and progresses slowly [36]. Cohen-Cymberknoh et al. studied the structural lung damage in PCD patients compared with CF patients using high-resolution CT, with the calculation of the total Brody scores (TBSs) [37]. The Brody score is a validated score for assessment of presence and severity of structural lung damage in patients with CF [38]. Using the TBS, they found that PCD lung disease was similar to CF pancreatic sufficient (CF-PS) (TBS of 30.8 for PCD vs. 31.4 for CF-PS) lung damage, but it differs in CF pancreatic insufficient (CF-PI) (TBS of 57.3, P, 0.001). Moreover, they found that the lower lobes are commonly involved in PCD as compared to CF; the upper lobes are usually spared in PCD. When they correlated the TBS with the forced expiratory volume at one second (FEV 1), they found that no correlation was seen between FEV 1 and TBS in PCD (r = 0.08, *p* = 0.71) [37]. It is important to note that our findings rely on a single CT scan, and for sure, further longitudinal studies are needed to precisely identify the radiological progression of the structural lung damage, the predictors of PCD structural lung damage, and the trajectories of spirometry measures.

Although it is estimated that about half of PCD patients have situs inversus (Kartagener’s syndrome) or other laterality defects, our study found a frequency of 36%. A total of 36% of situs inversus cases in our study were associated with the following genes: DNAH5, DNAI1, DNAI2, DNAAF5, and LRRC56. The lower prevalence of laterality defects in our study compared to what is known in the literature is likely due to the fact that a number of our patients had mutations in genes that affect the central pair or radial spoke components (RSPH1 and RSPH4), which do not cause left/right body asymmetry [1,4]. After stratifying the enrolled patients by laterality defects, we found that those with situs inversus had a higher PICARD score, a higher prevalence of NRD, and an earlier median age at diagnosis (2.8 years vs. 8.5 years). Other studies have also reported this trend. Kuehni et al. found that patients with PCD and situs inversus were diagnosed an average of 2 years earlier than those without situs inversus (3.5 years vs. 5.8 years; *p* = 0.001) [11]. A large study of 1375 children with PCD found that 30% were diagnosed within their first year of life: 52% of those with both situs anomalies and NRD, 33% of those with situs anomalies but no NRD, 21% of those with situs solitus and NRD, and 13% of those with situs solitus and no NRD [39]. Taken together, the presence of NRD and laterality defects in early infancy is a red flag for PCD, and clinicians should further evaluate these patients for a diagnosis of PCD.

The findings of this study have to be read in light of some limitations. First, with a small sample size and the absence of functional studies, caution must be applied; therefore, a larger sample size with incorporation of new cilia functional assessment diagnostic tools is required to validate and confirm the current findings. Second, the cross-sectional study design is a limiting factor to study the exact timing of the symptom’s appearance as well as the predictors of bronchiectasis. Nevertheless, these findings, for sure, would enriches the clinical phenotypes and genetic spectrum of PCD, particularly among Saudi patients, and provide more evidence for future genetic counseling and gene-targeted therapy for this disease. Furthermore, in SA, the national PCD registry is lacking, and the available data are only based on case series and case reports. Therefore, a national PCD registry is recommended.

## 5. Conclusions

To summarize, this study provides preliminary data on the clinical and genetic characteristics of PCD patients in the southwestern region of Saudi Arabia. We found that DNAH5 and RSPH9 genes were the most common genes among the studied population. Furthermore, PCD should be considered in children with early NRD and laterality defects, and further confirmatory tests are recommended. The study has limitations, but we suggest that further clinical and basic studies are needed to delineate the phenotype and genotype of PCD in this population. These findings also highlighting the need for greater awareness of the disease in daily clinical practice to facilitate early diagnosis and avoid irreversible lung damage.

## Figures and Tables

**Table 1:** Baseline characteristics of the patients.

Variable	n = 28
Age, median (IQR), y	7.5 (3–13)
Sex, male, No. (%)	14 (50)
Age at diagnosis, (IQR), y	5.5 (2–11)
Age at the first symptom appears, median (IQR), months	3 (1–6)
Neonatal respiratory distress, No. (%)	18 (64)
Duration of hospital stay, median (IQR), days	18 (11–42)
Family history of affected members, No. (%)	
- History of affected siblings	7 (25)
- History of other affected members	16 (57.1)
- History of consanguinity	26 (93)
Growth parameters	
- Weight, kg	18 (8–25)
- Height, cm	115 (78–128)
- Body mass index	15 (12–16)
- BMI percentile, median (IQR)	12 (3.5–46)
- BMI percentile < 5 percentile, No. (%)	8 (28.6)
PICADAR score	
- Median, (IQR)	8 (6–12)
- Minimum–maximum	4–14

IQR: interquartile range; No.: number; y: year; kg: kilograms; cm: centimeters; BMI: body mass index.

**Table 2:** Genetic variants of the patients (n = 28).

Family No.	Patient No.	Gene	DNA Change	Amino Acid Change	OMIM
1	1	RSPH9	c.804_806del	NA	612648
	2	RSPH9	c.804_806del	NA	612648
2	3	DNAH5	c.877dup	p.Arg293fs	NA
3	4	DNAI2	c.1631_1632insAGCG	NA	605483
	5	DNAI2	c.1631_1632insAGCG	NA	605483
	6	DNAI2	c.1631_1632insAGCG	NA	605483
4	7	DNAH5	c.6763C>T	p.Arg2255Ter	603335
	8	DNAH5	c.6763C>T	p.Arg2255Ter	603335
5	9	RSPH4A/SPAG1	c.1547C>T/c.1180G>A	p.Ala516Val/p.Glu394Lys	612647/603395
	10	RSPH4A/SPAG1	c.1547C>T/c.1180G>A	p.Ala516Val/p.Glu394Lys	612647/603395
6	11	SPEF2	c.3063G>C	p.Glu1021Asp	NA
7	12	DNAAF3	c.1513G>T	p.Gly505Ter	614566
8	13	TP73	c.1342G>A	p.Val448Met	601990
9	14	DNAH11/DNAH9	c.11839+1G>A/c.6457G>A	-/p.Ala2153Thr	603339/603330
10	15	RSPH4A	c.1547C>T	p.Ala516Val	612647
11	16	DNAH5	c.877dup	NA	NA
12	17	RSPH9	c.825G>C	p.Met275lle	612648
13	18	RSPH9	c.804_806del	p.K268del	612650
14	19	DNAAF5	c.2200delG	p.G734fs	614874
15	20	CCDC151/TP73	c.556A>G/c.1612C>T	p.Ser186Gly/p.Arg538Cys	615956/601990
16	21	DNAH11/DNAH9	c.4775G>T/c.3386G>T	p.Cys1592Phe/p.Ser1129Ile	603339/603330
17	22	DNAH5	c.877dup	p.Arg293fs	NA
18	23	DNAI1	c.1228G>A	p.G4105	244400
	24	DNAI1	c.1228G>A	p.G4105	244400
19	25	DNAI2	c.1408G>A	p.Gly470Ser	605483
20	26	LRRC56	c.494T>C	p.Leu165Pro	618254
	27	LRRC56	c.494T>C	p.Leu165Pro	618254
	28	LRRC56	c.494T>C	p.Leu165Pro	618254

**Table 3:** Chest radiograph and CT scan findings of the enrolled patients.

Variable	n = 28
Chest Radiograph findings, No. (%)	
- Lobar collapse/consolidation	19 (68)
- Bronchiole wall thickening	24 (86)
CT scan findings (n = 22), No. (%)	
- Bronchiectasis	17 (77)
- Mucus plugging	21 (95)
- Parenchymal changes of consolidation	22 (100)
- ground-glass density	8 (36)
Distribution of bronchiectasis (n = 17), No. (%)	
- Right-middle lobe	11 (65)
- Lingula	8 (47)
- Right upper lobes	5 (29)
- Left upper lobes	8 (47)
- Right lower lobes	15 (88)
- Left lower lobes	13 (76)

**Table 4:** Characteristics of primary ciliary dyskinesia in patients with situs solitus and situs inversus.

Variables	Situs Solitus n = 18 (64%)	Situs Inversus n = 10 (36%)	χ^2^/U	*p*-Value
Age at the time of study, median, y	9	5	69.5	0.324
Age at the time of diagnosis, median, y	8.5	2.8	58	0.124
Age at the first symptom appears, median, months	4	1	55	0.090
Neonatal respiratory distress, n	9	9	4.48	0.040
Duration of NICU admission, days	14	31.5	30	0.563
BMI percentile for age, median	10	15	56	0.269
PICADAR score, median	7.5	11.5	10.5	<0.001
Bronchiectasis, n	13	4	2.8	0.103

χ^2^: Chi-square test; U: Mann–Whitney U test; N: number; n: number; y: year; NICU: neonatal intensive care unit; BMI: body mass index. Statistical significance *p* < 0.05.

## Data Availability

On reasonable request, the corresponding author will provide the datasets used and/or analyzed during the current work.
